# Stable Lithium-Carbon Composite Enabled by Dual-Salt Additives

**DOI:** 10.1007/s40820-021-00633-3

**Published:** 2021-04-17

**Authors:** Lei Zheng, Feng Guo, Tuo Kang, Yingzhu Fan, Wei Gu, Yayun Mao, Ya Liu, Rong Huang, Zhiyun Li, Yanbin Shen, Wei Lu, Liwei Chen

**Affiliations:** 1 School of Nano-Tech and Nano-Bionics, University of Science and Technology of China, Hefei, 230026 People’s Republic of China; 2 i-Lab, CAS Center for Excellence in Nanoscience, Suzhou Institute of Nano-Tech and Nano-Bionics, Chinese Academy of Science, Suzhou, 215123 People’s Republic of China; 3 Vacuum Interconnected Nanotech Workstation (Nano-X), Suzhou Institute of Nano-Tech and Nano-Bionics (SINANO), Chinese Academy of Science (CAS), Suzhou, 215123 People’s Republic of China; 4 in-Situ Center for Physical Science, School of Chemistry and Chemical Engineering, Shanghai Jiaotong University, Shanghai, 200240 People’s Republic of China

**Keywords:** Lithium metal battery, Coulombic efficiency, Dual-salt additives, Li-CNT, Solid electrolyte interphase

## Abstract

Lithium metal is regarded as the ultimate negative electrode material for secondary batteries due to its high energy density. However, it suffers from poor cycling stability because of its high reactivity with liquid electrolytes. Therefore, continuous efforts have been put into improving the cycling Coulombic efficiency (CE) to extend the lifespan of the lithium metal negative electrode. Herein, we report that using dual-salt additives of LiPF_6_ and LiNO_3_ in an ether solvent-based electrolyte can significantly improve the cycling stability and rate capability of a Li-carbon (Li-CNT) composite. As a result, an average cycling CE as high as 99.30% was obtained for the Li-CNT at a current density of 2.5 mA cm^–2^ and an negative electrode to positive electrode capacity (N/P) ratio of 2. The cycling stability and rate capability enhancement of the Li-CNT negative electrode could be attributed to the formation of a better solid electrolyte interphase layer that contains both inorganic components and organic polyether. The former component mainly originates from the decomposition of the LiNO_3_ additive, while the latter comes from the LiPF_6_-induced ring-opening polymerization of the ether solvent. This novel surface chemistry significantly improves the CE of Li negative electrode, revealing its importance for the practical application of lithium metal batteries.

**Supplementary Information:**

The online version contains supplementary material available at 10.1007/s40820-021-00633-3.

## Highlights

- It is the first report that using dual-salt additives of LiPF_6_ and LiNO_3_ to significantly improve the cycling performance of the Li-CNT negative electrode.
- The mechanism why the combined use of LiPF_6_ and LiNO_3_ additive can improve the cycling performance and rate capability of the Li-CNT negative electrode was investigated.
- An average cycling Coulombic efficiency as high as 99.30% was obtained for the Li-CNT negative electrode at a current density of 2.5 mA cm^−2^ and an negative electrode to positive electrode capacity (N/P) ratio of 2.

**Supplementary Information:**

The online version contains supplementary material available at 10.1007/s40820-021-00633-3.

## Graphical abstract

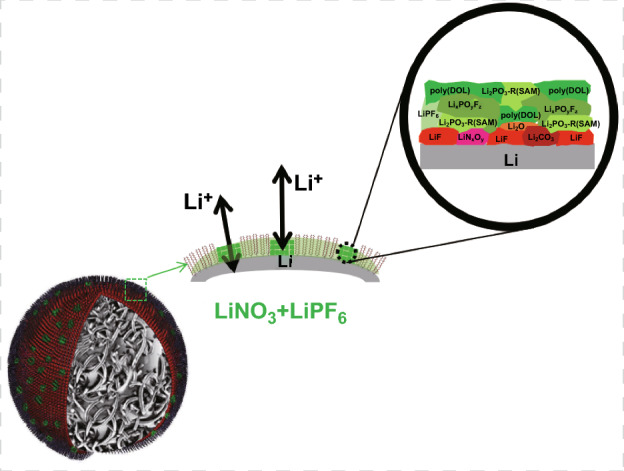

## Introduction

Lithium (Li) has been regarded as the ultimate negative electrode material for high energy density secondary batteries owing to its high energy density [1–7]. However, Li metal is highly reactive to electrolytes and is prone to the dendrite formation during the Li striping/plating cycling, resulting in limited cycle life and severe safety issue that largely impede the practical application of lithium metal batteries [5, 8–12]. Tremendous efforts have been dedicated to addressing the above problems in the last few decades, mainly including (1) regulating the homogeneity of Li deposition through controlling the negative electrode surface chemistry to suppress the dendrite formation [13–17], (2) employing 2D/3D hosts for the Li metal to increase the specific surface area of the negative electrode thus decrease the current density to suppress the dendrite formation [18–21], and (3) solidify the electrolyte or constructing a solid layer on Li surface to protect it from reacting with electrolytes [22–29].

**Fig. 1:**
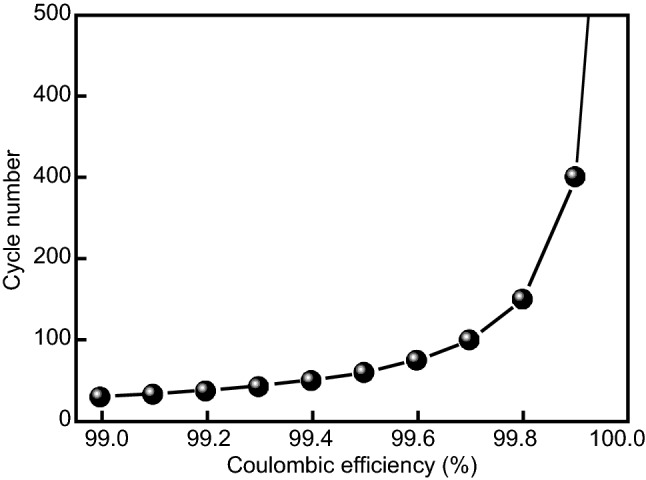
Calculated lifespan of a lithium metal anode based on its CE

Although many improvements in Li stability have been reported, typical average cycling Coulombic efficiency (CE) of Li metal in a commercial carbonate or ether solvent-based electrolyte at a practical current density (> 3 mA cm^−2^) is usually lower than 99.0% [30–35] (Table S1), which is far below the standard required for practical application. The lifespan of a lithium metal negative electrode is critically dependent on its CE (Fig. 1). Suppose that the end of life capacity retention is 80%, a lithium metal with an average cycling CE of 99.926% can sustain 300 cycles of Li plating/stripping, but the one with an average cycling CE of 99.0% can only sustain a lifespan less than 30 cycles. As a result, a large excess of lithium metal (negative electrode to positive electrode capacity (N/P) ratio > 50) is required to artificially enhance the cycle life, which brings down the volumetric energy density of the cell. Therefore, despite all the challenges, it is still crucial and worthwhile to push on the effort in improving the average cycling CE of lithium metal.

Recently, we reported a lithium-carbon nanotube (Li-CNT) microsphere composite with a hydrophobic self-assembled monolayer (SAM) surface passivation layer as an negative electrode material [36–42]. With the protection of the SAM layer, the Li-CNT composite exhibited an average cycling CE beyond 99.0% at a small N/P ratio of 2 [41]. However, the SAM layer could be damaged during Li plating/stripping cycles, which limited further improvement of CE [42].

In this work, we demonstrated the combined use of two additives, i.e., LiPF_6_ and LiNO_3_, in an ether-based electrolyte can generate a thin but robust solid electrolyte interphase (SEI) layer on Li-CNT when the SAM passivation layer was damaged, significantly improve the cycling stability and rate capability of the Li-CNT negative electrode. As a result, an average cycling CE as high as 99.30% was obtained for the Li-CNT composite negative electrode at a current density of 2.5 mA cm^−2^ and an N/P of 2.

## Experimental

### Material Synthesis and Li-CNT Electrode Preparation

The Li-CNT composite was synthesis according to our previous reports [37, 39–43]. 2 g of porous carbon nanotubes microspheres prepared by spray-drying were mixed with 4 g of Li in an Ar-filled glovebox at 180 °C for 20 min under stirring of 500 r min^−1^ [37, 41]. For the SAM passivation, 1 g of the Li-CNT was added to 10 mL of tetrahydrofuran (THF) solution containing 0.1 wt.% dihexadecanoalkyl phosphate (DHP) under stirring for 30 min. The resulting solution was vacuum-dried overnight to obtain the Li-CNT composite. To prepare a Li-CNT negative electrode, 20 mg of poly(styrene-co-butadiene) (Sigma-Aldrich), 20 mg of polystyrene (molecular weight: 2,000,000, Alfa Aesar), 40 mg of acetylene black, and 320 mg of Li-CNT composite were dissolved in a 1.5 mL p-xylene solvent (anhydrous, 99.5%, Sigma-Aldrich), and magnetically stirred for 4 h, then the slurry was coated on a copper foil, followed by vacuum-drying at 60 °C for 8 h in an Ar-filled glove box (O_2_ < 0.01 PPM, H_2_O < 0.01 PPM) [37, 41]. The areal capacity loading of the Li-CNT electrode can be controlled by varying the thickness of the electrode. In this work, the areal capacity loading of the Li-CNT electrode used in the Li-CNT||Li-CNT symmetric cell is 10 mA h cm^−2^. While in the Li-CNT||LFP cell, in order to obtain a low N/P ratio of 2, the areal capacity loading of the Li-CNT electrode is controlled to be 5 mAh cm^−2^. The deep-depth of discharge for the Li-CNT||Li-CNT symmetric cell and the Li-CNT||LiFePO_4_ full battery is 30% and 50%, respectively. All the Li-CNT||LFP cell was charging and discharging between 2.5–4.1 V.

### Physical and Chemical Characterizations

The morphology of the sample was characterized by scanning electron microscopy (SEM, Hitachi Regulus 8230) at 5 kV. Elemental composition was analyzed by energy-dispersive X-ray spectroscopy (EDS, Quanta FEG 250) operated at 20 kV. The X-ray photoelectron spectroscopy (XPS, PHI-5000 VersaProbe) of sample measurements all employed Al Kα radiation. The vibrational spectrum of SAM was characterized by sum-frequency generation (SFG) spectroscopy using an EKSPLA system with a copropagating configuration. The time-of-flight secondary ion mass spectrometry (TOF.SIMS5-100) was equipped with a 10 kV Bi^+^ ions analysis beam and 1 kV Cs^+^ sputtering for detecting negative secondary-ion fragments. The dimethyl carbonate was applied as a cleaning agent for cycled electrodes before the characterization.

### Electrochemical Characterizations

The 2025 button-type cells were assembled using Li-CNT as the negative electrode, polypropylene membrane (Celgard 2400) as the separator, lithium iron phosphate (LFP, Sinlion Battery Tech, Co., Ltd.) with an area capacity of 2.5 mAh cm^−2^ was used as the positive electrode, 1.0 M LiTFSI dissolved in a mixed solvent of DOL and DME (1:1 in volume) with 2 wt.% LiNO_3_ (Sigma-Aldrich) as the baseline electrolyte. The amount of electrolyte used was estimated at 60 μL for each cell. The battery charger system from NEWARE Technology Ltd., Shenzhen was used for the charging/discharging test. The electrochemical impedance spectroscopy was obtained on a VMP300 electrochemical workstation (Bio-Logic, France) in a frequency range from 100 to 1 MHz with an amplitude of 10 mV. Five parallel cells were used for each test.

## Results and Discussion

The Li-CNT composite was prepared by impregnating spray-dried CNT microparticles with molten Li to obtain a Li-CNT composite, which was subsequently passivated by a hydrophobic SAM layer of dihexadecanoalkyl phosphate (DHP) via a simple wet chemistry method [41, 42]. The SAM passivated Li-CNT composite has a micro-spherical shape with a diameter of about 5 µm (Fig. S1a). The successful preparation of the DHP SAM layer was verified by EDS, XPS, and SFG characterization (Fig. S1b–g). The SAM passivated Li-CNT composite shows a specific capacity of 1912 mAh g^−1^ at room temperature under a current density of 0.25 mA cm^−2^, and limited capacity loss (150 mAh g^−1^) was measured after storing in dry air (dew point: − 40 °C) for a week (Fig. S1h).

**Fig. 2:**
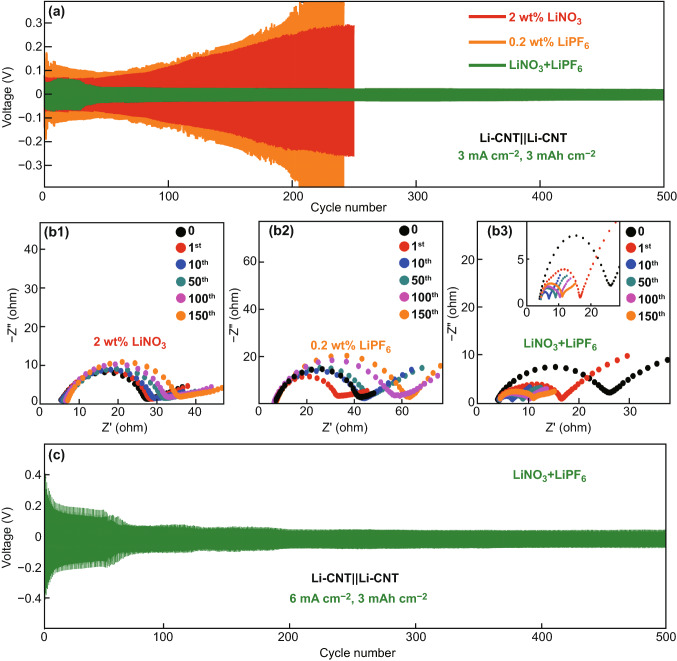
**a** Voltage profiles of Li-CNT||Li-CNT cells cycling at 3 mA cm^−2^, 3 mAh cm^−2^ in ether-based electrolytes with a single LiNO_3_ additive, a single LiPF_6_ additive, and dual-salt additives of LiPF_6_ and LiNO_3_. **b1**–**b3** Nyquist plots of the Li-CNT||Li-CNT cells showed at 3 mA cm^−2^, 3 mAh cm^−2^. **c** The voltage profiles of Li-CNT||Li-CNT cells cycling at 6 mA cm^−2^, 3 mAh cm^−2^ in ether-based electrolytes with dual-salt additives of LiPF_6_ and LiNO_3_

Li stripping/plating behaviors of SAM passivated Li-CNT (areal capacity loading: 10 mAh cm^−2^) symmetric cells with two different electrolytes are shown in Fig. 2. When using a typical ether-based electrolyte, that is, 1.0 M LiTFSI in a mixed solvent of DOL and DME (1:1 in volume), with 2 wt.% LiNO_3_ additive, the Li-CNT||Li-CNT cell shows stable Li stripping/plating for only ~ 60 cycles at a current density of 3 mA cm^−2^ and 3 mAh cm^−2^, and the overpotential quickly ascends, reaching 260 mV at the 250th cycle (Fig. 2a, red color). The overpotential of the Li-CNT||Li-CNT cell with a single LiPF_6_ additive is even larger than that with LiNO_3_ additive and also quickly ascends after ~ 50 cycles (Fig. 2a, orange color). In sharp contrast, the Li-CNT||Li-CNT symmetric cell with a dual-salt additives of LiPF_6_ and LiNO_3_ cycled at the same condition exhibits dramatically improved cycling performance with a significantly stabilized overpotential. As shown in Fig. 2a (green color), the overpotential of the Li-CNT||Li-CNT symmetric cell is around ~ 60 mV for the first 35 cycles and then decrease to ~ 40 mV that remains for 500 cycles, suggesting that the SEI layer formed during the initial cycles is a good conductor for Li-ions compared to that formed in the electrolyte with a single LiNO_3_ additive.

The impedance evolution of the Li-CNT||Li-CNT symmetric cells during cycling at a current density of 3 mA cm^−2^ was evaluated by the electrochemical impedance spectroscopy (EIS) measurement. As shown in Fig. 2b1, the impedance of the cell that cycled with a single LiNO_3_ additive increases gradually during cycling, rising from 28 to 36 Ω after 150 cycles, indicating the formation of ionic insulating SEI layer during cycling. Similar result is observed for that cell with a single LiPF_6_ additive (Fig. 2b2). While for the Li-CNT||Li-CNT symmetric cell cycled with dual-salt additives of LiPF_6_ and LiNO_3_, the impedance keeps decreasing during early cycles from 27 to 6 Ω and then maintains stable at around 10 Ω at 150 cycles (Fig. 2b3), indicating that an ion conductive and robust SEI layer is formed during earlier cycles. This result is in good agreement with the evolution of the overpotential showed in Fig. 2a.

Importantly, when the Li stripping/plating current density was further increased to 6 mA cm^−2^ (Fig. 2c), 8 mA cm^−2^ (Fig. S2a), and 10 mA cm^−2^ (Fig. S2b), the Li-CNT||Li-CNT symmetric cells cycled with dual-salt additives of LiPF_6_ and LiNO_3_ still show very stable cyclability with small overpotentials of 65, 118, and 217 mV, respectively, after an SEI formation activation stage at early cycles. These results further suggest that the SEI layer formed in the electrolyte with dual-salt additives of LiPF_6_ and LiNO_3_ is well ionic conductive and robust.

It is worth noting that the concentration of the LiPF_6_ additive was optimized by comparing the overpotential of the Li-CNT||Li-CNT symmetric cells. As shown in Fig. S2c, after 500 cycles under 3 mA cm^−2^, 3 mAh cm^−2^, cells cycled with 0, 0.1, 0.2, 0.3, 0.4, and 0.5 wt.% of the LiPF_6_ additive show the average overpotentials of 49, 48, 42, 46, 59, and 79 mV, respectively. Therefore, the optimized concentration of the LiPF_6_ additive was determined to be 0.2 wt.%.

**Fig. 3:**
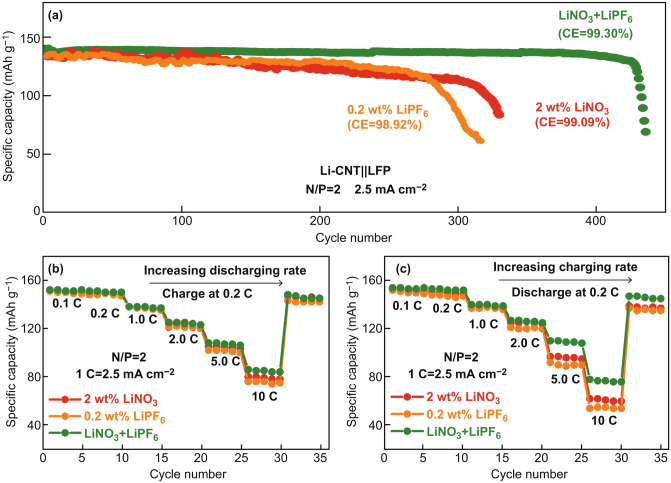
**a** Cycle performance of the Li-CNT||LFP batteries in ether-based electrolytes with a single LiNO_3_ additive, a single LiPF_6_ additive, and dual-salt additives of LiPF_6_ and LiNO_3_, **b**, **c** discharge rate ability and charge rate ability of the Li-CNT||LFP batteries

The average cycling CE of the Li-CNT negative electrode in the two different electrolytes was evaluated using a LiFePO_4_ positive electrode with a practical areal loading of 2.5 mAh cm^−2^ under a low N/P ratio of 2. As shown in Fig. 3a, the Li-CNT||LFP cell cycled with a single LiNO_3_ additive or a single LiPF_6_ additive at 1 C bewteen 2.5–4.1 V maintains stable for around 330 cycles and 270 cycles, respectively. before rapid capacity decay, corresponding to an average cycling CE of 99.09% and 98.92%, respectively. However, with dual-salt additives of LiPF_6_ and LiNO_3_, the cycle life of the Li-CNT||LFP cells can be further extended to 430 cycles, resulting in an average cycling CE of 99.30%, which is the best among reported literatures [37, 41, 42]. The voltage profile evolutions of the Li-CNT||LFP cells during cycling in ether-based electrolytes with different additives are showed in Fig. S3. Electrolyte decomposed product can be observed on the Li-CNT electrode in the Li-CNT||LFP cell after 200 cycles at 1 C in the in ether-based electrolytes with dual-salt additives of LiPF_6_ and LiNO_3_ (Fig. S4). However, the thickness of the Li-CNT electrode almost kept the same during cycling.

The Li-CNT||LFP cell cycled with dual-salt additives of LiPF_6_ and LiNO_3_ also shows improved rate performance, especially for the charge rate ability. As shown in Fig. 3b, when cells are charged at a constant current of 0.2 C and discharged at various C rates, the Li-CNT||LFP cell with dual-salt additives of LiPF_6_ and LiNO_3_ show slightly higher specific discharge capacities at 5 C and 10 C than those of the cell with only LiNO_3_ additive or LiPF_6_ additive; when the discharge current density is set at 0.2 C and the charge capacity varies (Fig. 3c), the Li-CNT||LFP cell with dual-salt additives of LiPF_6_ and LiNO_3_ shows even better charge capacities at 5 C and 10 C than that with a single LiNO_3_ or LiPF_6_ additive. These results again suggest that the SEI formed under the presence of both LiPF_6_ and LiNO_3_ additives has a smaller impedance for conducting the Li^+^ ions.

**Fig. 4:**
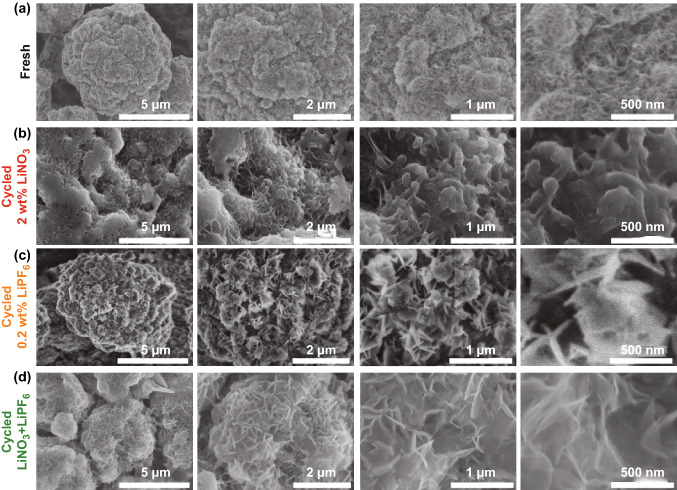
**a**–**d** SEM images of the Li-CNT electrode before (**a**) and after 50 cycles at a current density of 6 mA cm^−2^ in electrolytes with a single LiNO_3_ additive (**b**), LiPF_6_ additive (**c**), and dual-salt additives of LiPF_6_ and LiNO_3_ (**d**)

To probe why the Li-CNT SEI layer formed under the presence of dual-salt additives is more conductive and robust, morphology and chemistry evolution of the Li-CNT surface was analyzed by SEM, XPS, and TOF-SIMS. As shown in Fig. 4a, SEM images of the Li-CNT composite before cycling at various magnifications show a clear sponge-like CNT framework. After 50 cycles at a current density of 6 mA cm^−2^, the surface of the Li-CNT electrode cycled with a single LiNO_3_ additive or a single LiPF_6_ additive is covered with a thick and uneven deposited layer (Fig. 4b, c). However, the surface of the Li-CNT electrode cycled with dual-salt additives of LiPF_6_ and LiNO_3_ is covered with a unique thin surface layer composed of stacked-gossamer-like sheets (Fig. 4d), which could protect the lithium metal from the corrosion of the electrolyte and at the same time be a good conductor for Li-ion.

**Fig. 5:**
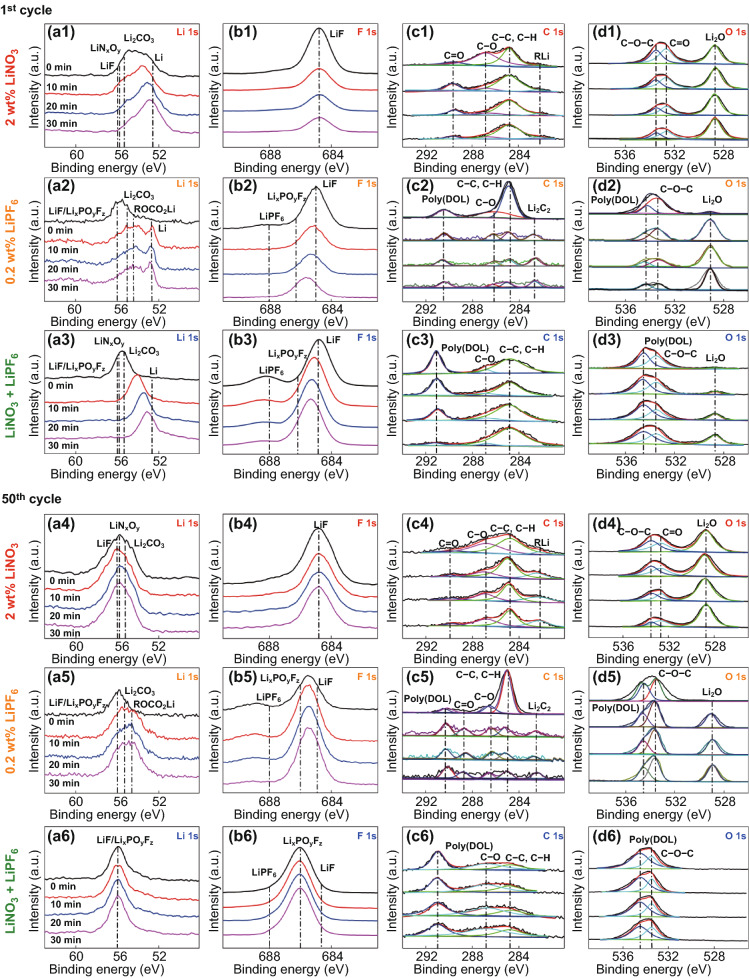
**a**–**d** Li 1s, F 1s, C 1s, and O 1s XPS depth profiles (etching time: 0 min, 10 min, 20 min, and 30 min) of the Li-CNT electrodes after different cycles in electrolytes with a single LiNO_3_ additive (**a1**–**d1, a4**–**d4**), LiPF_6_ additive (**a2**–**d2, a5**–**d5**), and dual-salt additives of LiPF_6_ and LiNO_3_ (**a3**–**d3, a6**–**d6**)

XPS depth profile analysis of the Li-CNT samples reveals very different surface chemistry evolution during cycling in electrolytes with a single LiNO_3_ additive and with dual-salt additives of LiPF_6_ and LiNO_3_. As shown in Fig. 5a1–d1, for the cell after the 1st cycle in the electrolyte with a single LiNO_3_ additive, the SEI layer on the surface of the Li-CNT electrode mainly composed of LiN_x_O_y_, Li_2_CO_3_, Li_2_O, and LiF, which could be originated from the decomposition of the LiTFSI salt, the ether solvent, and the LiNO_3_ additive [12, 44]. This layer is very thin at the 1st cycle and not uniformly covered on the surface as Li metal can be detected on the surface, and the peak intensity of SEI components decreases with the increasing of the depth (except the Li_2_O, which could come from the reaction of the Li-CNT with dissolved O_2_/CO_2_ in the electrolyte and decomposition of LiNO_3_ [44–46]). The thickness of the SEI layer grows fast with cycles while the components stay almost the same. The intensity of the Li metal detected on the surface of the Li-CNT negative electrode is much weaker at the 10th cycle (Fig. S5a1–d1) and disappears at the 50th cycle (Fig. 5a4–d4) (no signals even after the 30-min Ar sputtering), and the relative intensities of the LiN_x_O_y_, Li_2_CO_3_, Li_2_O, and LiF get stable after 10 cycles and present no obvious variation during the 30-min Ar sputtering. The SEI of the cell after the 1st cycle in the electrolyte with a single LiPF_6_ additive is also very thin and mainly composes LiF, Li_x_PO_y_F_z_, poly(DOL), organic carbonate, and Li_2_O (Fig. 5a2–d2). As shown in Fig. 5a5–d5, the relative content of the Li_x_PO_y_F_z_ and poly(DOL) increase, while that of the LiF decrease with cycling. The organic carbonate component seems only present on the top surface layer of the SEI.

The SEI formed on the Li-CNT cycled in the ether electrolyte with dual-salt additives of LiPF_6_ and LiNO_3_ exhibits distinct components. As shown in Fig. 5a3–d3, after the 1st cycle, the surface layer of the Li-CNT electrode includes the residual of LiPF_6_ (at 688 eV in the F 1s spectrum) [47] and its decomposition product Li_x_PO_y_F_z_ [26] (at 686.2 eV on the Li 1s spectrum), a small amount of LiF (684.8 eV on the F 1s spectrum), and prominent poly(DOL) signal (at 291 eV on the C 1 s and 534.5 eV on the O 1s spectra). This means that LiPF_6_ may induce the ring-opening polymerization of the DOL solvent to form polyether products [22]. The SEI layer is quite thin after the 1st cycle, as the intensity of the poly(DOL) decreases gradually during the Ar etching. However, the SEI grows and gets thick enough after the 10th (Fig. S5a2–d2) and 50th cycle (Fig. 5a6–d6), where no intensity variation can be observed for the SEI component during the 30-min Ar etching. The LiF/Li_x_PO_y_F_z_ components formed with the help of the LiPF_6_ additive could guarantee the mechanical strength and ionic conductivity of the SEI [26, 48, 49], while the organic polyether has better volume tolerance ability than inorganic salts components such as lithium alkyl and lithium carbonate that formed under a single LiNO_3_ additive [24, 50]. This could be the reason that a trace amount of LiPF_6_ additive can significantly improve the cycle performance of the Li-CNT negative electrode.

**Fig. 6:**
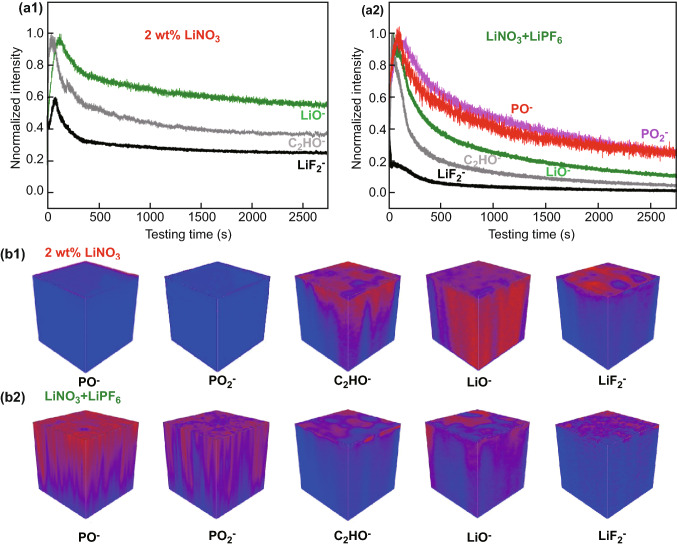
**a**, **b** TOF−SIMS depth profiles and distributions of PO^−^, PO_2_^−^, C_2_HO^−^, LiO^−^, and LiF_2_^−^ species on the surface of the Li-CNT electrodes after 50 cycles in electrolytes with a single LiNO_3_ additive (**a1**, **b1**) and dual-salt additives of LiPF_6_ and LiNO_3_ (**a2**, **b2**)

The different SEI chemistry of the Li-CNT electrodes after 50 cycles in ether-based electrolytes with a single LiNO_3_ additive and dual-salt additives of LiPF_6_ and LiNO_3_ has also been observed at the nano-scale by TOF-SIMS, which is a highly surface-sensitive technique with ultrahigh chemical selectivity. As shown in Fig. 6a2 and b2, a distinguishable PO^−^ and PO_2_^−^ signal appears in the Li-CNT electrode cycled with dual-salt additives of LiPF_6_ and LiNO_3_, which confirms the observation of the Li_x_PO_y_F_z_ component in the XPS spectra. The content of C_2_HO^−^ (representing organic SEI components that come from the solvent decomposition) on the Li-CNT electrode cycled with a single LiNO_3_ additive was relatively higher than that cycled with dual-salt additives of LiPF_6_ and LiNO_3_ (Fig. 6a1 and b1), indicating that the addition of LiPF_6_ additives can inhibit the decomposition of the organic solvents. Last but not the least, much higher LiO^−^ and LiF_2_^−^ signals can also be observed on the Li-CNT electrode cycled with a single LiNO_3_ additive, revealing the severe decomposition of the ether solvent and the LiTFSI salt.

## Conclusions

We demonstrated that the combined use of LiPF_6_ and LiNO_3_ additives in an ether-based electrolyte can significantly improve the cycling performance and rate capability of the Li-CNT negative electrode. The LiNO_3_ additive can generate inorganic products like LiN_x_O_y_, which have good mechanical strength, while the LiPF_6_ additive can help to induce the ring-opening polymerization of the DOL solvent to form polyether with good volumetric variation tolerance. These products together form an ionic conductive and robust SEI layer on the surface of the Li-CNT negative electrode. As a result, the Li-CNT||LFP cells with limited lithium excess (N/P = 2) has obtained an average cycling CE as high as 99.30% under a current density of 2.5 mA·cm^−2^.

## Supplementary Information

Below is the link to the electronic supplementary material.

Supplementary file1 (DOCX 1463 KB)
