# Supplementary material for: Stable Lithium-Carbon Composite Enabled by Dual-Salt Additives
Source: Nanomicro Lett. 2021 Apr 17;13:111. doi: 10.1007/s40820-021-00633-3 (PMC8053134; doi:10.1007/s40820-021-00633-3)
Supplement: Supplementary file 1 — Supplementary file1 (DOCX 1463 KB) [file 40820_2021_633_MOESM1_ESM.docx]

Supporting Information for

## Stable Lithium-Carbon Composite Enabled by Dual-Salt Additives

Lei Zheng^1,2^, Feng Guo^1,2^, Tuo Kang^2^, Yingzhu Fan^2^, Wei Gu^2^, Yayun Mao^2^, Ya Liu^2^, Rong Huang^3^, Zhiyun Li^3^, Yanbin Shen^2^,* Wei Lu^2^ and Liwei Chen^2,4^

^1^School of Nano-Tech and Nano-Bionics, University of Science and Technology of China, Hefei, 230026, China

^2^i-Lab, CAS Center for Excellence in Nanoscience, Suzhou Institute of Nano-Tech and Nano-Bionics, Chinese Academy of Science, Suzhou, 215123, China

^3^Vacuum Interconnected Nanotech Workstation (Nano-X), Suzhou Institute of Nano-Tech and Nano-Bionics (SINANO), Chinese Academy of Science (CAS), Suzhou 215123, China

^4^in-situ Center for Physical Science, School of Chemistry and Chemical Engineering, Shanghai Jiaotong University, Shanghai 200240, China

*Corresponding author. E-mail: ybshen2017@sinano.ac.cn.

# Supporting Tables and Figures

# 1. Supporting Methods

**Calculation of the average cycling Coulombic efficiency (CE):**

The method used to measure the average cycling Coulombic efficiency (CE) of the lithium metal negative electrode follows our previous papers [1-3]. The lithium carbon (Li-CNT) composite anode was paired with a highly reversible positive electrode, in this case, a commercial LiFePO_4_ (LFP) positive electrode is used for the estimation of CE. Suppose that the irreversible capacity loss of the LFP positive electrode during the cycling is ignored, and assume that all the capacity from the lithium negative electrode has been consumed at the plunge point in the cycling curve, then the CE of the negative electrode can be estimated from the initial capacity and the cycle number, as shown in the following equation:

CE=(C_positive_-C_total_/n)/ C_positive_

Where C_total_ is the total capacity of the positive electrode and negative electrode, C_positive_ is the positive electrode capacity, n is the cycle number at which the cycling curve starts to plunge.

For example, as shown in Fig. 3a, the initial cell capacity is 7.5 mA·h·cm^-2^ (negative electrode: 5.0 mA·h·cm^-2^, positive electrode: 2.5 mA·h·cm^-2^), and the capacity retention curve of the Li-CNT||LFP cell cycled at 1 C starts to plunge at around 330 cycles, so the CE of Li-CNT can be estimated to be 99.09%.

CE= $\frac{2.5-\frac{7.5}{330}}{2.5}$= 99.09%

**Table S1.** Summary of average cycling Coulombic efficiency of Li metal in a commercial ether or carbonate solvent-based electrolytes.

| **Reference** | **Electrolytes** | **Type** | **Current density (mA**·**cm^-2^)** | **Capacity**  **density (mA**·**h**·**cm^-2^)** | **Cycle number** | **Average CE(%)** |
| --- | --- | --- | --- | --- | --- | --- |
| [4] | 1 M LiTFSI in DOL/DME (1:1 v/v) + 1 wt. % LiNO_3_ + PDMS-OCH_3_ | Li||Cu | 0.5 | 1 | 200 | 97 |
|  |  | Li\|\|Cu | **3** | **3** | **70** | **95.4** |
| [5] | 1 M LiTFSI in DOL/DME (1:1 v/v) + 1 wt. % LiNO_3_ | Li@3D Cu||LFP | 0.5 | 0.5 | 1200 | 98.2 |
| [6] | 1 M LiTFSI in DOL/DME (1:1 v/v) + 2 wt. % LiNO_3_ | Li||Cu | 0.5 | 0.5 | 200 | 99.1 |
| [7] | 1 M LiTFSI in DOL/DME (1:1 v/v) + 2 wt. % LiNO_3_ | Li||LFP | 0.2 | 1 | 50 | 98.6 |
|  |  |  | 2 | 1 | 50 | 97.7 |
| [8] | 1 M LiTFSI in DOL/DME (1:1 v/v) + 1 wt. % LiNO_3_ | Li||NG@Cu | 1 | 1 | 200 | 98 |
| [9] | 1 M LiTFSI in DOL/DME (1:1 v/v) | Li||Li | 1 | 2 | 50 | 98 |
|  |  |  | 1 | 5 | 50 | 97 |
|  |  |  | 2 | 1 | 100 | 92 |
|  |  |  | 0.2 | 1 | 100 | 95 |
| [10] | 1 M LiTFSI in DOL/DME (1:1 v/v) | Li||Li | 2 | 2 | 300 | 98.8 |
| [11] | 1 M LiTFSI in DOL/DME (1:1 v/v) + 2 wt. % LiNO_3_ | Li||C_3_N_4_@Ni | 2 | 4 | 100 | 98 |
|  |  |  | 0.5 | 1 | 300 | 98 |
| [12] | 1 M LiFSI in OFE/DME | Li||Cu | 1 | 1 | 1000 | 95 |
| [13] | 1 M LiTFSI + 1 M LiFSI in DME | Li||Cu | 0.5 | 0.5 | 200 | 98.2 |
| [14] | 1 M LiTFSI in DOL/DME (1:1 v/v) + 1 wt. % LiNO_3_ | Li||ESM@Cu | 1 | 1 | 200 | 98 |
|  |  |  | 2 | 1 | 200 | 97 |
|  |  |  | **3** | **1** | **170** | **97** |
|  |  |  | **5** | **1** | **150** | **96** |
| [15] | 1 M LiTFSI in DOL/DME (1:1 v/v) | Li||coated Cu | 1 | 3 | 140 (25 ℃) | 97.6 |
|  |  |  | 1 | 3 | 55 (50 ℃) | 98.5 |
| [16] | 1 M LiFSI-3TMS) in DME (1:1 v/v) | Li||Cu | 0.5 | 1 | 150 | 98.8 |
| [17] | 1 M LiTFSI in DOL/DME (1:1 v/v) | Li||PDA@Cu | 2 | 1 | 150 | 96.4 |
| [18] | 1 M LiTFSI in DOL/DME (1:1 v/v) + LiNO_3_ | Li||G@POF | 1 | 1 | 300 | 98 |
|  |  |  | 2 | 2 | 150 | 98 |
|  |  |  | **3** | **3** | **50** | **95** |
| [19] | 1 M LiTFSI in DOL/DME (1:1 v/v) | Li||coiled Cu | **5** | **1** | **150** | **92** |
| [20] | 1 M LiTFSI in DOL/DME (1:1 v/v) + 1 wt. % LiNO_3_ | Li||3DCMP | 1 | 1 | 100 | 97.6 |
|  |  |  | **4** | **1** | **35** | **94.6** |
| [21] | 1 M LiTFSI in DOL/DME (1:1 v/v) + THU | Li||Cu | 0.5 | 1 | 350 | 98.5 |
|  |  |  | **5** | **1** | **120** | **96** |
| [22] | 1 M LiPF_6_ in EC/DEC (1:1 v/v) + 0.15 M LiDFP | Li||Cu | 0.4 | 0.5 | 100 | 95.2 |
| [23] | 1 M LiPF_6_ in EC/DEC (1:1 v/v) + Glass fiber | Li||Cu | 1 | 2 | 217 | 92.7 |
| [24] | 0.5 M LiPF_6_ in EC/DEC (1:1 v/v) + LiNO_3_ | Li||Cu | 1 | 1 | 200 | 98.1 |
| [25] | 1 M LiPF_6_ in EC/DEC (1:2 v/v) + 2 wt. % TTFEB | Li||Cu | 0.1 | 0.5 | 100 | 99 |
| [26] | 1 M LiPF_6_ in EC/DMC/DEC (1:1:1 v/v/v) + 8 wt. % AlCl_3_ | Li||Cu | 0.5 | 2 | 150 | 98 |
| [27] | LiFSI/DMC/TTE (1:1.5:1.5 n/n/n) | Li||Cu | 1 | 1 | 400 | 98.6 |
| [28] | 1 M LiPF_6_ in EC/DMC/DME (3.5:3.5:3 v/v/v) | Li||Cu | 0.1 | 0.5 | 100 | 96 |

**2. Supporting Figures**


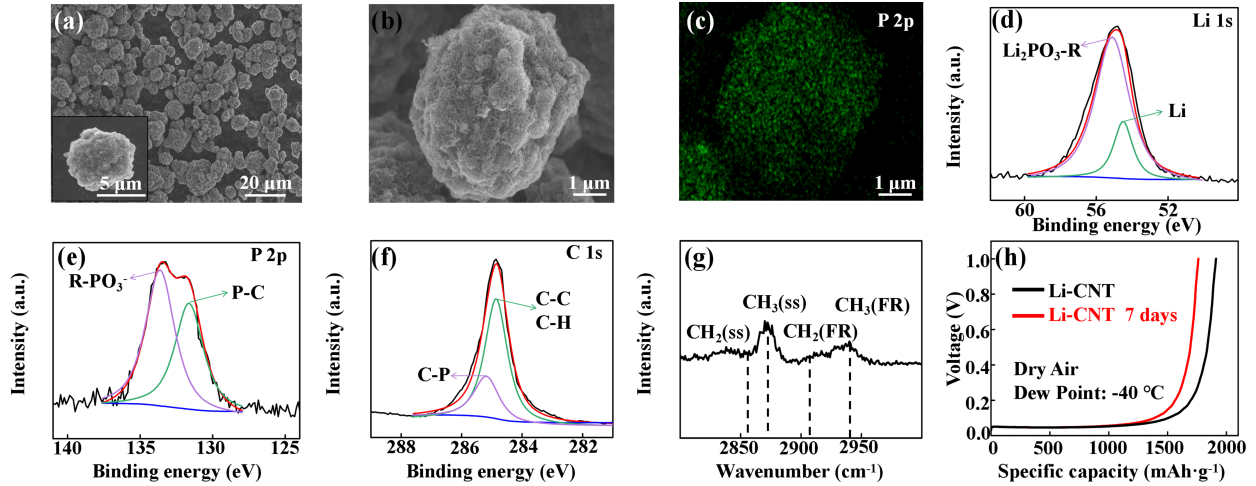


**Fig. S1** SEM images of Li-CNT samples (a), SEM and elemental mapping of the SAM passivated Li-CNT composite particle (b, c). XPS spectra of the passivated Li-CNT composite: (d) Li 1s spectrum, (e) P 2p spectrum, (f) C 1s spectrum. SFG vibrational spectrum of the passivated Li-CNT microsphere (g) and galvanostatic discharge curves of the passivated and dry-air exposed Li-CNT samples (h).

The Li-CNT composite has a microspherical shape with a diameter of about 5 µm (Fig. S1a) [1-2], and the hydrophobic self-assembled monolayer (SAM) layer of dihexadecanoalkyl phosphate (DHP) was assembled on the surface of the Li-CNT composite [3], which could be detected by the EDS mapping through phosphor element (Fig. S1b and S1c). XPS characterization results (Li 1s, P 2p, C 1s) of the passivated Li-CNT composite show obvious peaks at 55.3 and 54.5 eV in the Li 1s spectrum (Fig. S1d) that can be assigned to lithium phosphate and Li, respectively, [29-30] peaks at 133.5 and 131.5 eV in the P 2p spectrum (Fig. S1e) that can be assigned to the P in the P-O and P-C bonds, respectively [31], and peaks at 285.0 and 284.8 eV in the C 1s spectrum (Fig. S1f) are usually assigned to the C in C-P, C-C, or C-H bonds [32], confirming the existing of lithium alkyl phosphate molecule on the surface of the Li-CNT composite. SFG vibrational spectrum obtained from the passivated Li-CNT sample (Fig. S1g) shows methylene symmetric stretching (CH_2_(ss)) at 2855 cm^-1^, methyl symmetric stretching (CH_3_(ss)) at 2881 cm^-1^, and their corresponding methylene Fermi resonance modes (CH_2_(FR)) at 2910 cm^-1^ and methyl Fermi resonance (CH_3_(FR)) at 2940 cm^-1^, respectively, evidencing the monolayer structure of the DHP passivated layer [33-34]. The passivated Li-CNT shows a specific capacity of 1912 mA·h·g^-1^ at room temperature under a current density of 0.25 mA·cm^-2^, and limited capacity loss (150 mA·h·g^-1^) was measured after storing in dry air (dew point: -40 °C) for a week (Fig. S1h), indicating that the SAM layer has effectively protected the Li metal from corroding by the air.


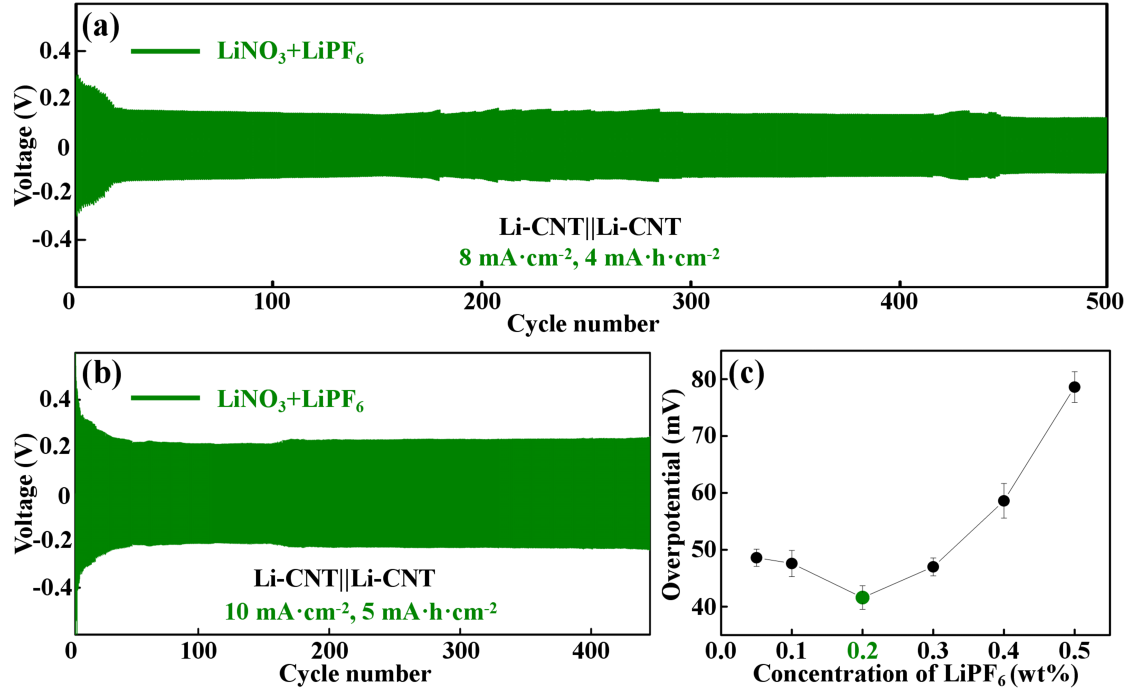


**Fig. S2** The voltage profiles of Li-CNT||Li-CNT cells cycling at 8 mA·cm^-2^, 4 mA·h·cm^-2^ (a) and 10 mA·cm^-2^, 5 mA·h·cm^-2^ (b) in ether-based electrolyte with dual-salt additives of LiPF_6_ and LiNO_3_. Overpotential comparison of the slurry-coated Li-CNT||Li-CNT cells after 500 cycles in ether-based electrolyte with 2 wt. % LiNO_3_ and different concentrations of LiPF_6_ additive under 3 mA·cm^-2^, 3 mA·h·cm^-2^ (c).

**
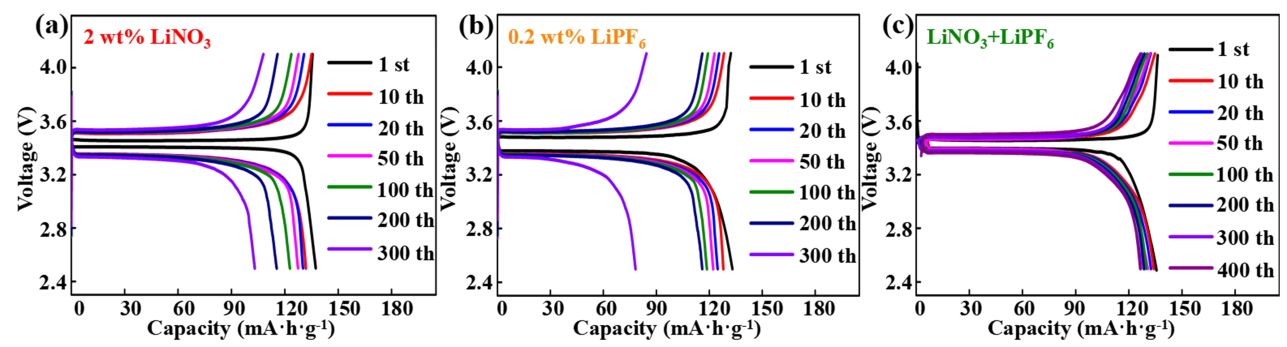
**

**Fig. S3** Voltage profile evolutions of the Li-CNT||LFP cells during cycling in ether-based electrolytes with different additives at 1 C in the range of 2.5-4.1 V.

**
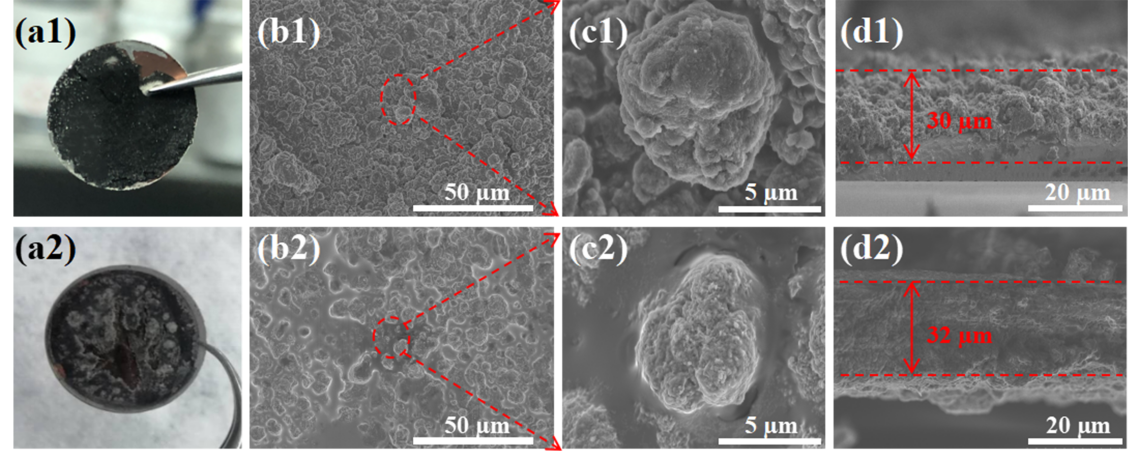
**

**Fig. S4** Morphology of the Li-CNT electrode in the Li-CNT||LFP cell before (a1-d1) and after 200 cycles (a2-d2) at 1 C in the in ether-based electrolytes with dual-salt additives of LiPF_6_ and LiNO_3_.


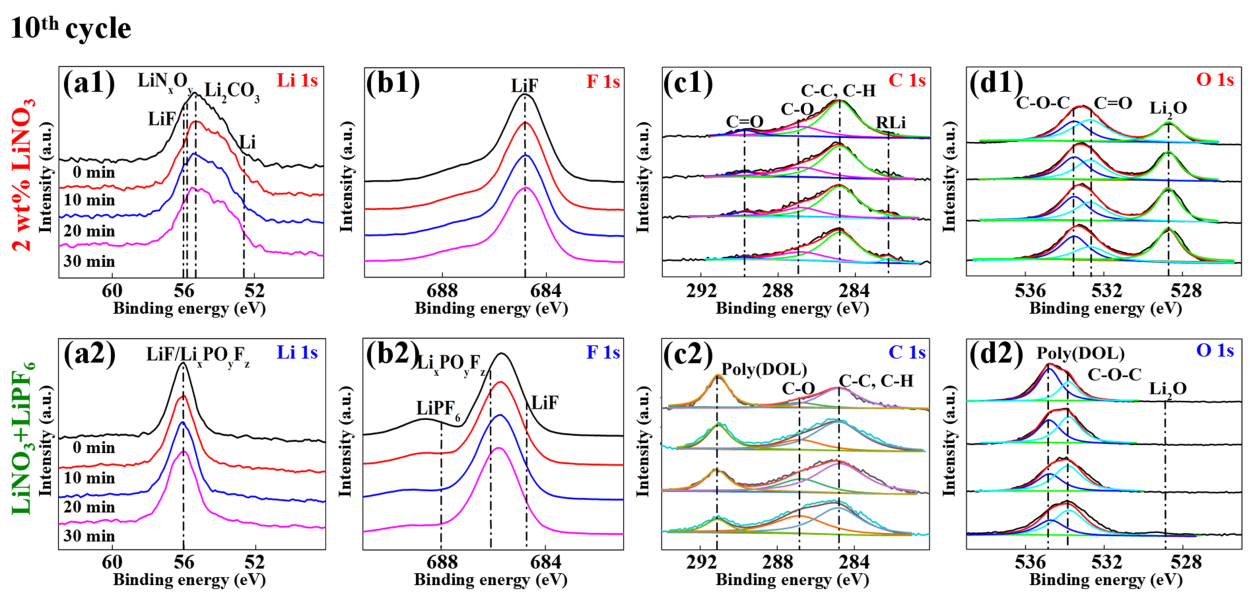


**Fig. S5** Li 1s, F 1s, C 1s and O 1s XPS depth profiles (etching time: 0 min, 10 min, 20 min and 30 min) of the Li-CNT electrodes after 10 cycles in electrolytes with a sole LiNO_3_ additive (a1-d1) and dual-salt additives of LiPF_6_ and LiNO_3_ (a2-d2).

**Supplementary References**

[S1] Y. L. Wang, Y. B. Shen, Z. L. Du, X. F. Zhang, K. Wang, H. Y. Zhang, T. Kang, F. Guo, C. H. Liu, X. D. Wu, L. Wei, L. W. Chen. A lithium-carbon nanotube composite for stable lithium anodes. J. Mater. Chem. A **5**, 23434-23439 (2017). <https://doi.org/>10.1039/c7ta08531a

[S2] T. Kang, Y. L. Wang, F. Guo, C. B. Liu, J. H. Zhao, J. Yang, H. Z. Lin, Y. J. Qu, Y. B. Shen, W. Lu, L. W. Chen. Self-assembled monolayer enables slurry-coating of li anode. ACS Cen. Sci. **5**, 468-476 (2019). <https://doi.org/10.1021/acscentsci.8b00845>

[S3] L. Zheng, F. Guo, T. Kang, J. Yang, Y. Liu, W. Gu, Y. F. Zhao, H. Z. Lin, Y. B. Shen, W. Lu, L. W. Chen. Highly stable lithium anode enabled by self-assembled monolayer of dihexadecanoalkyl phosphate. Nano Res. **26**, (2019). <https://doi.org/10.1007/s12274-019-2565-7>

[S4] J. Meng, F. Chu, J. Hu, C. Li. Liquid polydimethylsiloxane grafting to enable dendrite‐free li plating for highly reversible li‐metal batteries. Adv. Funct. Mater. **29**, (2019). <https://doi.org/10.1002/adfm.201902220>

[S5] P. Li, X. Dong, C. Li, J. Liu, Y. Liu, W. Feng, C. Wang, Y. Wang, Y. Xia. Anchoring an artificial solid-electrolyte interphase layer on a 3d current collector for high-performance lithium anodes. Angew. Chem. Int. Edit. **58**, 2093-2097 (2019). <https://doi.org/10.1002/anie.201813905>

[S6] Y. Han, Y. Jie, F. Huang, Y. Chen, Z. Lei, G. Zhang, X. Ren, L. Qin, R. Cao, S. Jiao. Enabling stable lithium metal anode through electrochemical kinetics manipulation. Adv. Funct. Mater. **29**, (2019). <https://doi.org/10.1002/adfm.201904629>

[S7] J. Qian, B. D. Adams, J. Zheng, W. Xu, W. A. Henderson, J. Wang, M. E. Bowden, S. Xu, J. Hu, J.-G. Zhang. Anode-free rechargeable lithium metal batteries. Adv. Funct. Mater. **26**, 7094-7102 (2016). <https://doi.org/10.1002/adfm.201602353>

[S8] R. Zhang, X. R. Chen, X. Chen, X. B. Cheng, X. Q. Zhang, C. Yan, Q. Zhang. Lithiophilic sites in doped graphene guide uniform lithium nucleation for dendrite-free lithium metal anodes. Angew. Chem. Int. Edit. **56**, 7764-7768 (2017). <https://doi.org/10.1002/anie.201702099>

[S9] L. Ma, M. S. Kim, L. A. Archer. Stable artificial solid electrolyte interphases for lithium batteries. Chem. Mater. **29**, 4181-4189 (2017). <https://doi.org/10.1021/acs.chemmater.6b03687>

[S10] H. Duan, J. Zhang, X. Chen, X. D. Zhang, J. Y. Li, L. B. Huang, X. Zhang, J. L. Shi, Y. X. Yin, Q. Zhang, Y. G. Guo, L. Jiang, L. J. Wan. Uniform nucleation of lithium in 3d current collectors via bromide intermediates for stable cycling lithium metal batteries. J. Am. Chem. Soc. **140**, 18051-18057 (2018). <https://doi.org/10.1021/jacs.8b10488>

[S11] Z. Lu, Q. Liang, B. Wang, Y. Tao, Y. Zhao, W. Lv, D. Liu, C. Zhang, Z. Weng, J. Liang, H. Li, Q.-H. Yang. Graphitic carbon nitride induced micro-electric field for dendrite-free lithium metal anodes. Adv. Energy Mater. **9**, (2019). <https://doi.org/10.1002/aenm.201803186>

[S12] J. Zheng, G. Ji, X. Fan, J. Chen, Q. Li, H. Wang, Y. Yang, K. C. DeMella, S. R. Raghavan, C. Wang. High‐fluorinated electrolytes for li–s batteries. Adv. Energy Mater. **9**, (2019). <https://doi.org/10.1002/aenm.201803774>

[S13] J. Alvarado, M. A. Schroeder, T. P. Pollard, X. Wang, J. Z. Lee, M. Zhang, T. Wynn, M. Ding, O. Borodin, Y. S. Meng, K. Xu. Bisalt ether electrolytes: A pathway towards lithium metal batteries with ni-rich cathodes. Energ. Environ. Sci. **12**, 780-794 (2019). <https://doi.org/10.1039/c8ee02601g>

[S14] Z. Ju, J. Nai, Y. Wang, T. Liu, J. Zheng, H. Yuan, O. Sheng, C. Jin, W. Zhang, Z. Jin, H. Tian, Y. Liu, X. Tao. Biomacromolecules enabled dendrite-free lithium metal battery and its origin revealed by cryo-electron microscopy. Nat. Commun. **11**, 488 (2020). <https://doi.org/10.1038/s41467-020-14358-1>

[S15] Y. T. Weng, H. W. Liu, A. Pei, F. Shi, H. Wang, C. Y. Lin, S. S. Huang, L. Y. Su, J. P. Hsu, C. C. Fang, Y. Cui, N. L. Wu. An ultrathin ionomer interphase for high efficiency lithium anode in carbonate based electrolyte. Nat. Commun. **10**, 5824 (2019). <https://doi.org/10.1038/s41467-019-13783-1>

[S16] X. Ren, S. Chen, H. Lee, D. Mei, M. H. Engelhard, S. D. Burton, W. Zhao, J. Zheng, Q. Li, M. S. Ding, M. Schroeder, J. Alvarado, K. Xu, Y. S. Meng, J. Liu, J.-G. Zhang, W. Xu. Localized high-concentration sulfone electrolytes for high-efficiency lithium-metal batteries. Chem. **4**, 1877-1892 (2018). <https://doi.org/10.1016/j.chempr.2018.05.002>

[S17] J. Jiang, Z. Pan, Z. Kou, P. Nie, C. Chen, Z. Li, S. Li, Q. Zhu, H. Dou, X. Zhang, J. Wang. Lithiophilic polymer interphase anchored on laser-punched 3d holey cu matrix enables uniform lithium nucleation leading to super-stable lithium metal anodes. Energy Storage Mater. **29**, 84-91 (2020). <https://doi.org/10.1016/j.ensm.2020.04.006>

[S18] B. Q. Li, X. R. Chen, X. Chen, C. X. Zhao, R. Zhang, X. B. Cheng, Q. Zhang. Favorable lithium nucleation on lithiophilic framework porphyrin for dendrite-free lithium metal anodes. Research **2019**, 4608940 (2019). <https://doi.org/10.34133/2019/4608940>

[S19] Y. Chen, M. Yue, C. Liu, H. Zhang, Y. Yu, X. Li, H. Zhang. Long cycle life lithium metal batteries enabled with upright lithium anode. Adv. Funct. Mater. **29**, (2019). <https://doi.org/10.1002/adfm.201806752>

[S20] X.-Y. Hu, P. Xu, S. Deng, J. Lei, X. Lin, Q.-H. Wu, M. Zheng, Q. Dong. Inducing ordered li deposition on a pani-decorated cu mesh for an advanced li anode. J. Mater. Chem. A **8**, 17056-17064 (2020). <https://doi.org/10.1039/d0ta03929b>

[S21] Q. Wang, C. Yang, J. Yang, K. Wu, C. Hu, J. Lu, W. Liu, X. Sun, J. Qiu, H. Zhou. Dendrite-free lithium deposition via a superfilling mechanism for high-performance li-metal batteries. Adv. Mater. **31**, 190-248 (2019). <https://doi.org/10.1002/adma.201903248>

[S22] P. C. Shi, L. C. Zhang, H. F. Xiang, X. Liang, Y. Sun, W. Xu. Lithium difluorophosphate as a dendrite-suppressing additive for lithium metal batteries. ACS Appl. Mater. Inter. **10**, 22201-22209 (2018). <https://doi.org/10.1021/acsami.8b05185>

[S23] Q. W. Shi, Y. R. Zhong, M. Wu, H. Z. Wang, H. L. Wang. High-capacity rechargeable batteries based on deeply cyclable lithium metal anodes. P. Natl. Acad. Sci. USA **115**, 5676-5680 (2018). <https://doi.org/10.1073/pnas.1803634115>

[S24] Y. Y. Liu, D. C. Lin, Y. Z. Li, G. X. Chen, A. Pei, O. Nix, Y. B. Li, Y. Cui. Solubility-mediated sustained release enabling nitrate additive in carbonate electrolytes for stable lithium metal anode. Nat. Commun. **9**, (2018). <https://doi.org/> 10.1038/s41467-018-06077-5

[S25] Y. L. Ma, Z. X. Zhou, C. J. Li, L. Wang, Y. Wang, X. Q. Cheng, P. J. Zuo, C. Y. Du, H. Huo, Y. Z. Gao, G. P. Yin. Enabling reliable lithium metal batteries by a bifunctional anionic electrolyte additive. Energy Storage Mater. **11**, 197-204 (2018). <https://doi.org/10.1016/j.ensm.2017.10.015>

[S26] H. Ye, Y. X. Yin, S. F. Zhang, Y. Shi, L. Liu, X. X. Zeng, R. Wen, Y. G. Guo, L. J. Wan. Synergism of al-containing solid electrolyte interphase layer and al-based colloidal particles for stable lithium anode. Nano Energy **36**, 411-417 (2017). <https://doi.org/10.1016/j.nanoen.2017.04.056>

[S27] N. Piao, X. Ji, H. Xu, X. L. Fan, L. Chen, S. F. Liu, M. N. Garaga, S. C. Greenbaum, L. Wang, C. S. Wang, X. M. He. Countersolvent electrolytes for lithium-metal batteries. Adv. Energy Mater. **10**, (2020). <https://doi.org/10.1002/aenm.201903568>

[S28] H. L. Yu, J. N. Zhao, L. B. Ben, Y. J. Zhan, Y. D. Wu, X. J. Huang. Dendrite-free lithium deposition with self aligned columnar structure in a carbonate-ether mixed electrolyte. ACS Energy Lett. **2**, 1296-1302 (2017). <https://doi.org/10.1021/acsenergylett.7b00273>

[S29] W. E. Morgan, W. J. Stec, J. R. Vanwazer. Inner-orbital photoelectron spectroscopy of alkali-metal halides, perchlorates, phosphates, and pyrophosphates. J. Am. Chem. Soc. **95**, 751-755 (1973).

[S30] J. P. Contour, A. Salesse, M. Froment, M. Garreau, J. Thevenin, D. Warin. Analysis by electron-microscopy and xps of lithium surfaces polarized in anhydrous organic electrolytes. J. Microsc. Spect. Elec. **4**, 483-491 (1979).

[S31] Y. M. Shulga, A. V. Bulatov, R. A. T. Gould, W. V. Konze, L. H. Pignolet. X-ray photoelectron-spectroscopy of a series of heterometallic gold platinum phosphine cluster compounds. Inorg. Chem. **31**, 4704-4706 (1992). <https://doi.org/0020-166919211331-4704$03.00/0>

[S32] J. Swiatowska, V. Lair, C. Pereira-Nabais, G. Cote, P. Marcus, A. Chagnes. Xps, xrd and sem characterization of a thin ceria layer deposited onto graphite electrode for application in lithium-ion batteries. Appl. Surf. Sci. **257**, 9110-9119 (2011). <https://doi.org/10.1016/j.apsusc.2011.05.108>

[S33] S. Roke, O. Berg, J. Buitenhuis, A. van Blaaderen, M. Bonn. Surface molecular view of colloidal gelation. P. Natl. Acad. Sci. USA **103**, 13310-13314 (2006). <https://doi.org/> <http://www.jstor.org/stable/2687809>

[S34] H. Zhang, F. J. Li, Q. B. Xiao, H. Z. Lin. Conformation of capping ligands on nanoplates: Facet-edge-induced disorder and self-assembly-related ordering revealed by sum frequency generation spectroscopy. J. Phys. Chem. Lett. **6**, 2170-2176 (2015). <https://doi.org/10.1021/acs.jpclett.5b00717>
